# Low-molecular-weight fucoidan inhibits the proliferation of melanoma via Bcl-2 phosphorylation and PTEN/AKT pathway

**DOI:** 10.32604/or.2023.044362

**Published:** 2023-12-28

**Authors:** MINJI PARK, CHULHWAN BANG, WON-SOO YUN, YUN-MI JEONG

**Affiliations:** 1 T&R Biofab Co., Ltd., Seongnam-si, 13487, Korea; 2 Department of Dermatology, Seoul St. Mary’s Hospital, College of Medicine, The Catholic University of Korea, Seoul, 02706, Korea; 3 Department of Mechanical Engineering, Tech University of Korea, Si-heung City, 15073, Korea

**Keywords:** Low-molecular-weight fucoidan, Melanoma, Patient-derived melanoma explants in a 3D-printed collagen scaffold, Anti-melanoma effect, PTEN-AKT-Bcl-2 network

## Abstract

Fucoidan, a sulfate polysaccharide obtained from brown seaweed, has various bioactive properties, including anti-inflammatory, anti-cancer, anti-viral, anti-oxidant, anti-coagulant, anti-thrombotic, anti-angiogenic, and anti-*Helicobacter pylori* properties. However, the effects of low-molecular-weight fucoidan (LMW-F) on melanoma cell lines and three dimensional (3D) cell culture models are not well understood. This study aimed to investigate the effects of LMW-F on A375 human melanoma cells and cryopreserved biospecimens derived from patients with advanced melanoma. Ultrasonic wave was used to fragment fucoidan derived from *Fucus vesiculosus* into smaller LMW-F. MTT and live/dead assays showed that LMW-F inhibited cell proliferation in both A375 cells and patient-derived melanoma explants in a 3D-printed collagen scaffold. The PTEN/AKT pathway was found to be involved in the anti-melanoma effects of fucoidan. Western blot analysis revealed that LMW-F reduced the phosphorylation of Bcl-2 at Thr 56, which was associated with the prevention of anti-apoptotic activity of cancer cells. Our findings suggested that LMW-F could enhance anti-melanoma chemotherapy and improve the outcomes of patients with melanoma resistance.

## Introduction

According to projections from the American Cancer Society, in 2023 an estimated 97,610 new cases of melanoma will be diagnosed in the United States, with men accounting for 68,120 cases and women 30,490 [1,2]. Additionally, it is expected that approximately 7,990 deaths will occur due to melanoma, with men accounting for 5,420 of these deaths and women accounting for 2,570 [1,2]. Advanced melanoma therapy encompasses a range of treatments that aim to manage and potentially cure melanoma that has metastasized to other parts of the body [3,4]. These therapies include surgery, radiation therapy, chemotherapy, immunotherapy, targeted therapy, and combination therapies. The choice of treatment is based on several factors, including the stage and location of the cancer, the overall health of the patient, and the specific genetic mutations driving the cancer’s growth [3–5]. Despite significant advances, limitations persist in the field of advanced melanoma therapy. Approximately half of all patients are unresponsive to cancer immunotherapies. In addition, targeted therapies may initially result in major tumor reduction in most patients, but these treatments become ineffective in the subsequent weeks or months for at least three-quarters of those patients [5,6]. Furthermore, medications used in advanced melanoma therapy can cause adverse effects that in turn may impact the patient’s quality of life [5,6]. To overcome these limitations, current research on treating advanced melanoma primarily focuses on hit drug discovery.

Fucoidans are sulfated polysaccharides found in brown algae species such as *Fucus vesiculosus* and *Ascophyllum nodosum* [7,8]. They are complex mixtures of polysaccharides containing L-fucose as the primary monosaccharide component and may or may not contain fucan sulfate [7,8]. Fucoidan has emerged as a research hotspot due to its potential biological properties, including anti-oxidant, anti-inflammatory, anti-tumoral, anti-viral, and anti-diabetic effects [7,8]. Fucoidans are typically extracted using methods such as water baths, acid baths, or microwave heating, and the bioactivity of fucoidan varies depending on the extraction method [8]. In particular, low-molecular-weight fucoidan (LMW-F) is a smaller molecular weight form of fucoidan that has improved solubility and bioavailability compared to regular fucoidan [9]. LMW-F has been reported to inhibit cell growth and proliferation in several cancer cell lines [10–12], but the underlying cellular mechanism of its anti-melanoma effects on 2D and 3D cell culture models remains unclear. The present study aimed to investigate the effects of LMW-F on the viability and proliferation of A374 cells and the growth and survival of patient-derived melanoma explants (PDMEs) in a 3D-printed collagen scaffold (3D-PCS-PDME) *in vitro* system. Additionally, we investigated the precise mechanisms by which LMW-F may trigger anti-proliferation-related signaling pathways and apoptosis pathways, including the AKT pathway, Bcl-2 expression, and caspase-3 expression.

## Materials and Methods

### Reagents, cell lines, and 3D-PCS-PDMEs

The fucoidan from *Fucus vesiculosus* was purchased from Sigma (St. Louis, MO, USA). Live and dead viability/cytotoxicity kits and MTT assay kits were purchased from Thermo Fisher Scientific (Rockford, IL, USA). MS Collagen (type 1 atelo-collagen from porcine skin) was obtained from MS Bio, Inc. (Gyeonggi, Korea). Antibodies recognizing p-Bcl-2 (Ser70) (sc-293128), Bcl-2 (sc-7382), H2B (sc-515808), caspase-3 (SC-56052) and actin (SC-58673) were obtained from Santa Cruz Biotechnology, Inc. (Santa Cruz, CA, USA). Antibodies recognizing p-AKT (Ser473) (#9271), p-PTEN (Ser380) (#9551), p-ERK (#9101), p-p38 (#9211), p-JNK (#9251), and p-Bcl-2 (Thr56) (#2875) were obtained from Cell Signaling Technology (MA, USA). Apoptosis western blot cocktail (pro/p17-caspase-3, cleaved PARP1, muscle actin) (ab136812) was obtained from Abcam (Cambridge, UK). The A735 human malignant melanoma cell lines were purchased from American Type Culture Collection (ATCC, Rockville, MD, USA). The human fibroblast cell line CCD-986 SK cells were obtained from the Korea Cell Line Bank (KCLB, Seoul, Korea). The A375 and CCD-986 SK cells were cultured in complete medium [DMEM-high glucose supplemented with 10% FBS and 1% PS (penicillin-streptomycin)]. To assess the dose-dependent effects of LMW-F on A375 cell viability, cells were treated with LMW-F at concentrations ranging from 0, 1, 5, 10, 20, 50 μg/mL and incubated for 24 h. Cell viability was then assessed using a live/dead assay and MTT assay, as described previously [13–15]. 1–2 mm PDME fragments were obtained from patient-derived melanoma xenograft models, which were sourced from Seoul St. Mary’s Hospital (Seoul, Korea), as described in our prior studies [13–15].

### Ultrasound assisted preparation of LMW-F stock concentrations

We prepared stock concentrations of LMW-F (10 and 50 mg/mL) using a modified ultrasonication method and PBS buffer, as previously described [8,9]. LMW-F was dissolved in PBS buffer and treated in an ultrasonic bath (DATHAN Scientific, Korea) under conditions of 60 kHz frequency, 25°C temperature, 30 min time, 117 W power, 100% amplitude, and sweep mode. The LMW-F stock concentrations were further diluted for subsequent experiments.

### 3D-PCS-PDME fabrication for culture and LMW-F treatment

3D-PCS-PDMEs were prepared as previously described [13,14]. Uniform and stable 3D-PCSs were manufactured using an extrusion-based 3D printing method with an X Printer (T&R Biofab Co., Ltd., Gyeonggi-do, Korea). 3D-PCS-PDMEs were cultured in a-Minimum Essential Medium with 10% fetal calf serum, 2 mM L-glutamine, and 1% PS in the presence or absence of LMW-F in a dose-dependent manner. They were then incubated for 14 days at 37°C in a 5% CO_2_ incubator.

### Cell viability and proliferation assays

Cell viability and proliferation assays were conducted using a live and dead assay and MTT assay, as previously described [13–15]. Absorbance of MTT assay was measured using a spectrometer (Emax; Molecular Devices, Sunnyvale, CA, USA). To further assess the viability of A375 cells, a double staining kit of calcein-acetoxymethyl (AM) and propidium iodide (PI) was used to fluorescently label live and dead cells, respsectively [13,14]. Cells were incubated at 37°C in a 5% CO_2_ incubator for 30 min with protection from light. Images were acquired using an Olympus FV1200 confocal microscope with laser lines at 405, 473, 559, and 635 nm.

### H&E staining

The H&E staining protocol was performed as Formalin-fixed paraffin-embedded tissue sections were deparaffinized and rehydrated through xylene and graded alcohol washes [13]. The sections were then immersed in Harris hematoxylin solution for a duration of approximately 5–10 min to facilitate nuclear staining. Following hematoxylin staining, the slides were differentiated using acid alcohol or Scott’s tap water for a few seconds. The slides were rinsed with distilled water thereafter. For cytoplasmic and extracellular staining, the slides were treated with eosin Y solution for a brief period of 1–5 min, and subsequently rinsed with distilled water. Dehydration of the slides was achieved through sequential immersion in increasing concentrations of ethanol (70%, 95%, and 100%). Finally, clearing of the tissue sections was accomplished using xylene or a xylene substitute. Following dehydration and clearing, coverslips were mounted onto the tissue sections using DPX mountant. The mountant was allowed to dry, and the edges of the coverslips were sealed using clear nail polish or a similar sealant. The slides were then ready for observation and analysis under a light microscope.

### Masson’s trichrome (MT) staining

Sample sections were deparaffinized and subsequently rehydrated via successive washes in xylene and graded alcohols. Following these preparations, the sections were immersed in Weigert’s iron hematoxylin solution, imparting a deep blue-black hue to the nuclei. To achieve optimal staining contrast, an acid-alcohol rinse was then performed, effectively facilitating the differentiation of nuclei. Subsequently, the sections were stained in a solution comprising Biebrich scarlet-acid fuchsin, which imparts a vivid red coloration to the cytoplasm and muscle fibers. To ensure specificity in highlighting the connective tissue, a phosphomolybdic acid solution was applied, selectively binding to collagen fibers. This acid solution enhances the staining precision by accentuating the collagen fibers within the tissue. Lastly, counterstaining was performed using aniline blue solution, effectively coloring the background and muscle fibers. Aniline blue demonstrates preferential affinity towards collagen fibers and the extracellular matrix, resulting in their conspicuous blue appearance. The stained sections were subsequently subjected to dehydration, clearing, and finally mounted with coverslips, enabling microscopic examination.

### Western blot analysis

The samples were disrupted using a homogenizer, after which an ice-cold RIPA protein extraction solution with a protease inhibitor cocktail (iNtRON biotechnology, Inc., Seoul, Korea) was added, and the samples were homogenized with stainless steel beads (Qiagen, Cam, USA). Protein concentrations were assessed using a BCA-kit (Thermo Scientific, Rockford, IL, USA). An equal amount of protein (50 μg) from each sample was loaded onto 10% to 12% SDS gel, and transferred to a PVDF membrane (Merk Millipore, MA, USA). The membranes were blocked for 2 h at room temperature with 5% nonfat dry milk in PBS containing 0.1% Tween-20 and incubated with anti-bodies (1:1000) overnight at 4°C (Suppl. Table 1). After washing three times, the membranes were incubated with a horseradish peroxidase-conjugated secondary antibody (1:5000) at RT for 2 h and visualized with a chemiluminescence substrate. Quantitative analysis of the western blot images was performed using ImageJ Software.

## Results

### LMW-F inhibits the proliferation of A375 melanoma cells in a dose-dependent manner

**Figure 1:**
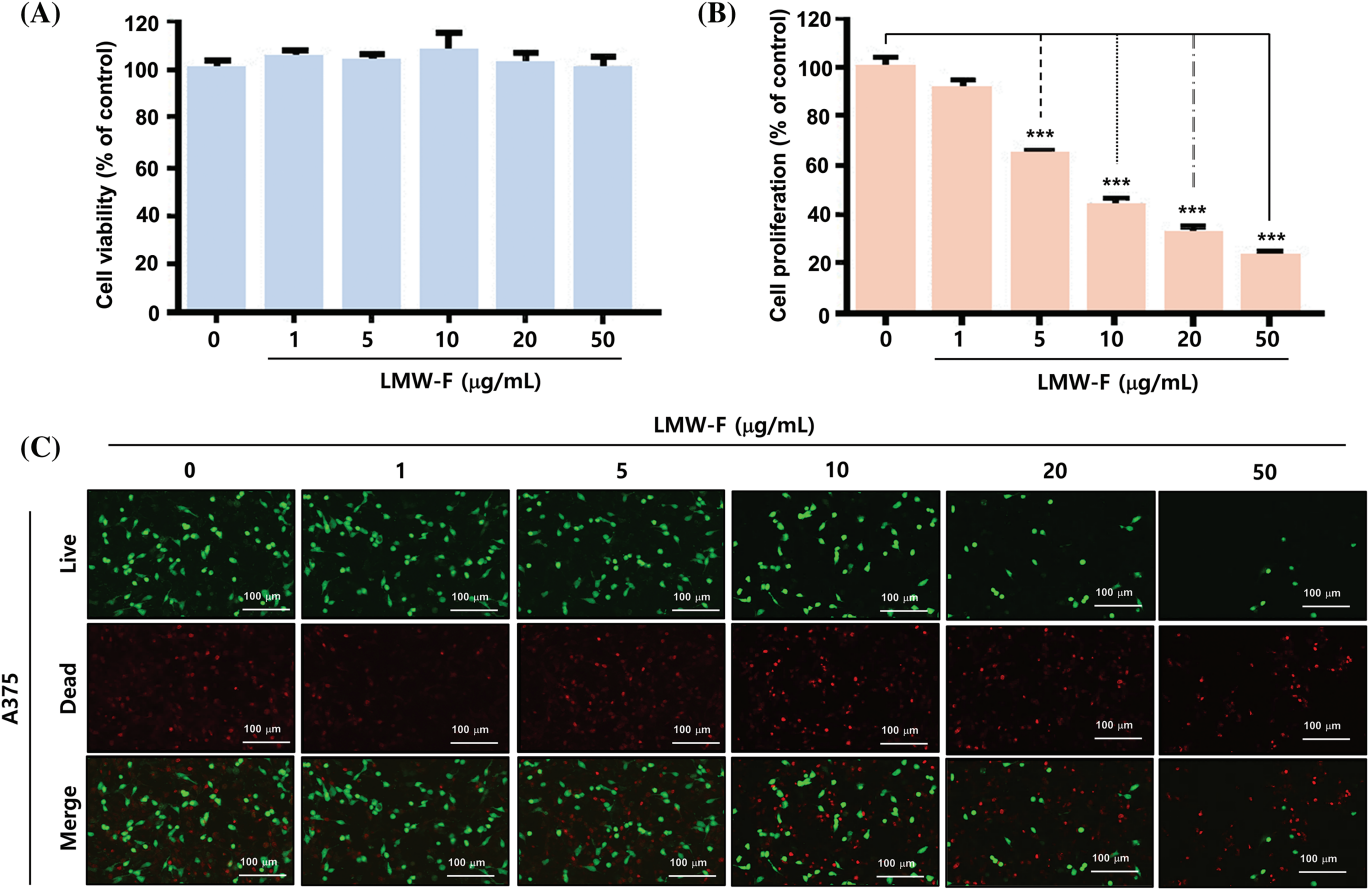
LMW-F inhibits the cell proliferation of A375 cell line. (A) Dose-dependent cell viability of LMW-F-treated A375 cells. (B) Dose-dependent cell proliferation of LMW-F-treated A375 cells. A375 cells were treated with LMW-F (0 to 50 μg/mL) and incubated for 24 h to assess cell viability or 72 h to evaluate cell proliferation. The bar graph represents triplicate assays and is expressed as a percentage of the untreated group. Data is presented as the mean ± SD and was analyzed using Student’s *t*-test. ***p < 0.001 *vs.* corresponding controls. (C) Confocal live images show dose-dependent effects of LMW-F treatment on cell proliferation in A375 cells, independently indicating live cells (green) and dead cells (red). Scale bars represent 100 μm.

To investigate the effects of LMW-F on melanoma cell viability and proliferation, we treated A375 cells with LMW-F at concentrations ranging from 0 to 50 μg/mL for 24 and 72 h. We evaluated cell viability and proliferation using MTT assays and live/dead staining. Treatment with 50 μg/mL LMW-F did not affect A375 cell viability (Fig. 1A). However, treatment with 5 μg/mL LMW-F resulted in a 40% inhibition of cell proliferation, while treatment with 50 μg/mL LMW-F led to an approximately 80% inhibition of cell proliferation (Fig. 1B). These effects of LMW-F on A375 cell viability and proliferation were confirmed by live/dead staining (Fig. 1C). To examine potential dose-dependent effects of LMW-F on normal cells, we treated CCD-986 SK fibroblast with various concentrations of LMW-F for 24 and 72 h. Our results showed that LMW-F treatment did not affect CCD-986 SK fibroblast viability or proliferation in a dose-dependent manner (Suppl. Fig. S1), indicating that LMW-F has an anti-proliferation effect on A375 melanoma cells. These findings suggested that LMW-F inhibits the proliferation of A375 melanoma cells in a dose-dependent manner, while having no significant effect on the viability or proliferation of CCD-986 SK fibroblasts.

### LMW-F downregulates the PTEN/AKT signaling pathway and Bcl-2 phosphorylation at Thr 56

**Figure 2:**
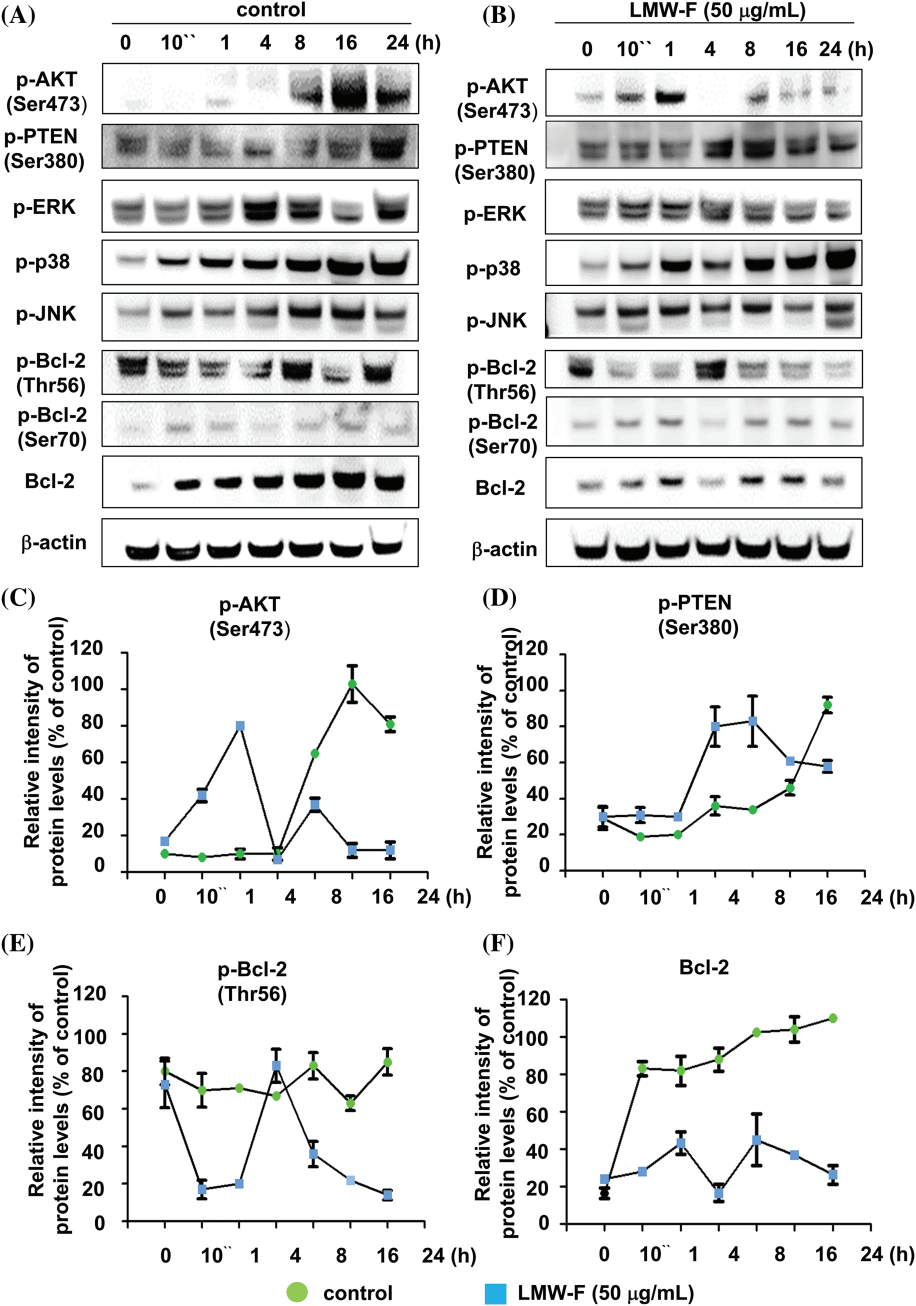
LMW-F negatively affects phosphorylation of Bcl-2 at Thr56 and PTEN/AKT pathway. A375 cells were treated with LMW-F (50 μg/mL) at the indicated time points and harvested for western blot analysis of targeted signaling pathways, as described in more detail in the Materials and Methods section. (A, B) Western blot analysis indicates the phosphorylation of representative targeted signaling pathways. (C–F) The line graphs depict the results from quantitative analysis of the western blot images. Each data point indicates the band intensity measured by a densitometer (control, green circle; LMW-F (50 μg/mL), blue square). β-actin was used as an internal control to normalize protein expression. Data are expressed as the mean ± SD of triplicate assays, relative to control. Statistical analysis was performed using Student’s *t*-test. ***p < 0.001 *vs.* corresponding controls.

To investigate the effects of LMW-F on proliferation-related signaling pathways in melanoma cells, we performed western blot analyses to detect key cellular pathways, including AKT, ERK, p38, and JNK, which are important signaling transduction pathways, and types of protein kinases in cells (Figs. 2A–2F). Untreated A375 cells exhibited AKT phosphorylation at Ser473 for at least 8 h. The phosphorylation of PTEN at Ser473 increased for 24 h, whereas LMW-F related phosphorylation of PTEN at Ser380 decreased after 16 h (Figs. 2A–2D). Treatment with LMW-F in A375 cells induced phosphorylation of AKT at Ser475 after 10 min, followed by increased phosphorylation at 1 h. After 4 h, LMW-F related phosphorylation of AKT at Ser473 was downregulated, and phosphorylation of PTEN at Ser380 increased (Figs. 2B–2D). These findings might be because phosphorylation of PTEN at Ser380 regulates PTEN stability and AKT dephosphorylation at (Ser473), consequently suppressing tumorigenesis [16,17]. In the presence of LMW-F, the PTEN pathway at Ser380 negatively regulates the AKT pathway (Figs. 2A, 2C, and 2D). Treatment with LMW-F slightly affects the EKR, p38, or JNK pathways compared to untreated A375 cells (Suppl. Figs. S2 and S3). Furthermore, LMW-F treatment downregulated the phosphorylation of Bcl-2 at Thr56 and the expression of Bcl-2 (Figs. 2B, 2E, and 2F). Phosphorylation of Bcl-2 at Ser70 has been associated with cell survival and proliferation by enhancing the anti-apoptotic activity of Bcl-2, whereas phosphorylation of Bcl-2 at Thr56 has been reported to play a role in promoting apoptosis [18,19]. These results suggest that LMW-F may inhibit cell proliferation and promote apoptosis in melanoma cells by regulating PTEN/AKT signaling and Bcl-2 phosphorylation.

### Inhibitory impact of LMW-F on the outgrowth of 3D-PCS-PDME

**Figure 3:**
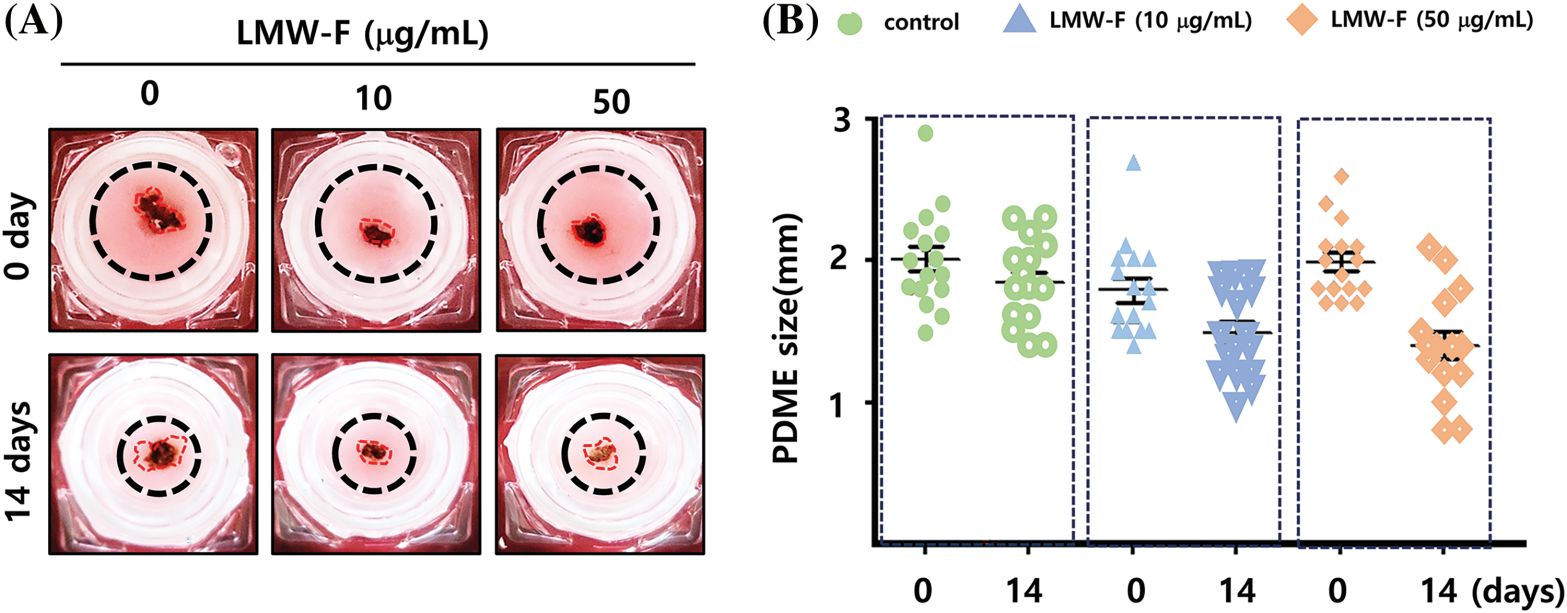
Outgrowth of 3D-PCS-PDMEs in the control group and dose-dependent treatment of LMW-F under long-term culture conditions. (A) Representative images show the morphological changes of 3D-PCS-PDMEs under untreated conditions or with LMW-F treatment (0, 10, 50 μg/mL) at 0 and 14 days. (B) The graph shows the size of the PDMEs for each culture condition (0, green; 10 μg/mL LMW-F, blue; 50 μg/mL LMW-F, orange) at 0 and 14 days of incubation. The statistical analysis of the data provides evidence of dose-dependent morphological outgrowths of PDME in the presence of LMW-F.

**Figure 4:**
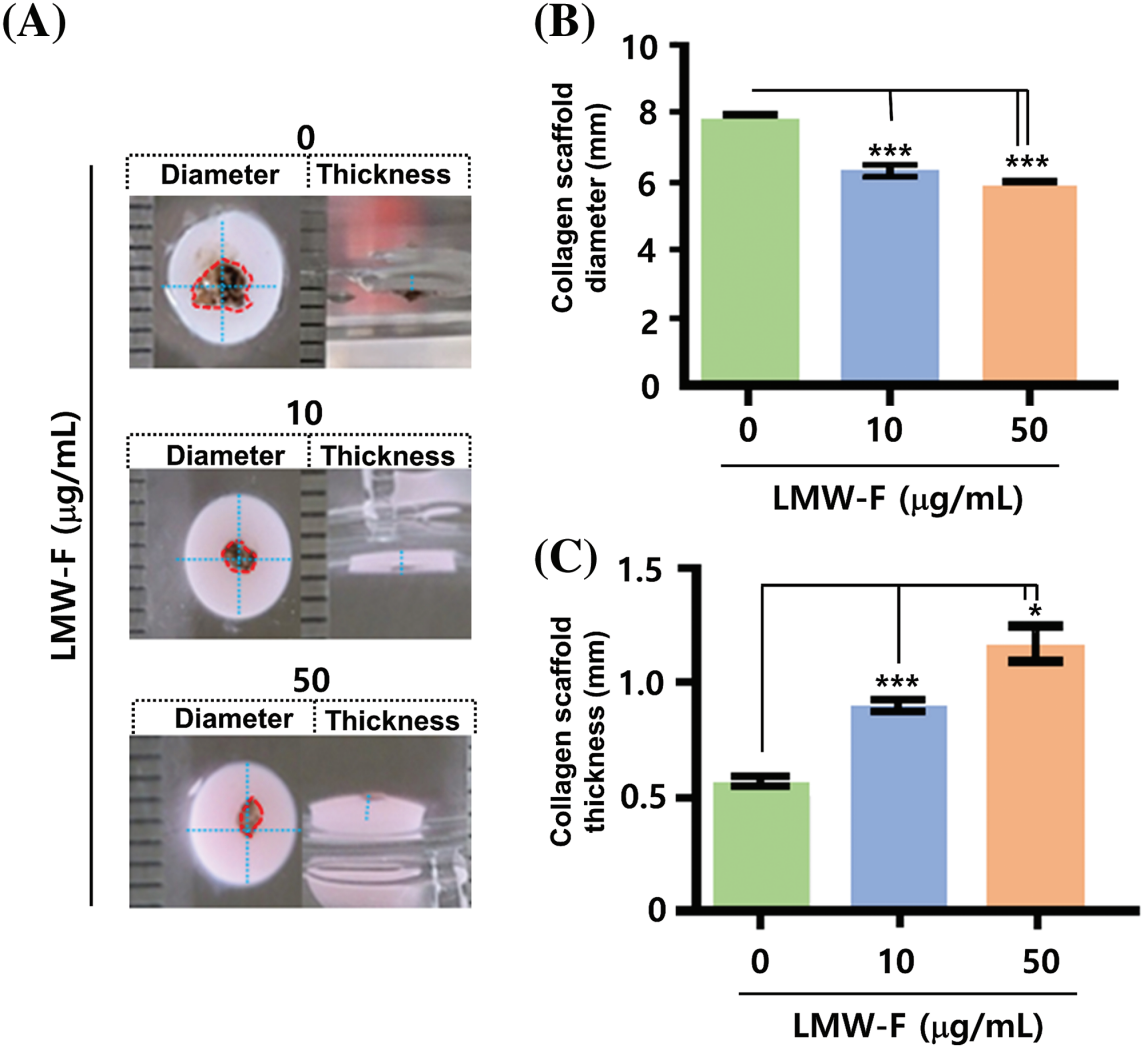
Effects of LMW-F on diameter and thickness in 3D-PCS-PDMEs under long-term conditions. (A) Representative images show the morphological changes of 3D-PCS-PDMEs after LMW-F treatment (0, 10, 50 μg/mL) for 14 days. The diameter and thickness of the 3D-PCSs are highlighted with a blue dashed line, while the PDME fragments are indicated with a red dashed line. (B) The graph shows the diameter of 3D-PCSs in the presence or absence of LMW-F. (C) The graph shows the thickness of 3D-PCSs in the presence or absence of LMW-F. The diameter and thickness were measured using a ruler. Scale bars, 1 mm. All data were obtained from three independent experiments. Data represent the mean ± SD of triplicate assays expressed as a percentage of the untreated group. Data were analyzed using Student’s *t*-test. *p < 0.05, ***p < 0.001 *vs.* corresponding controls.

**Figure 5:**
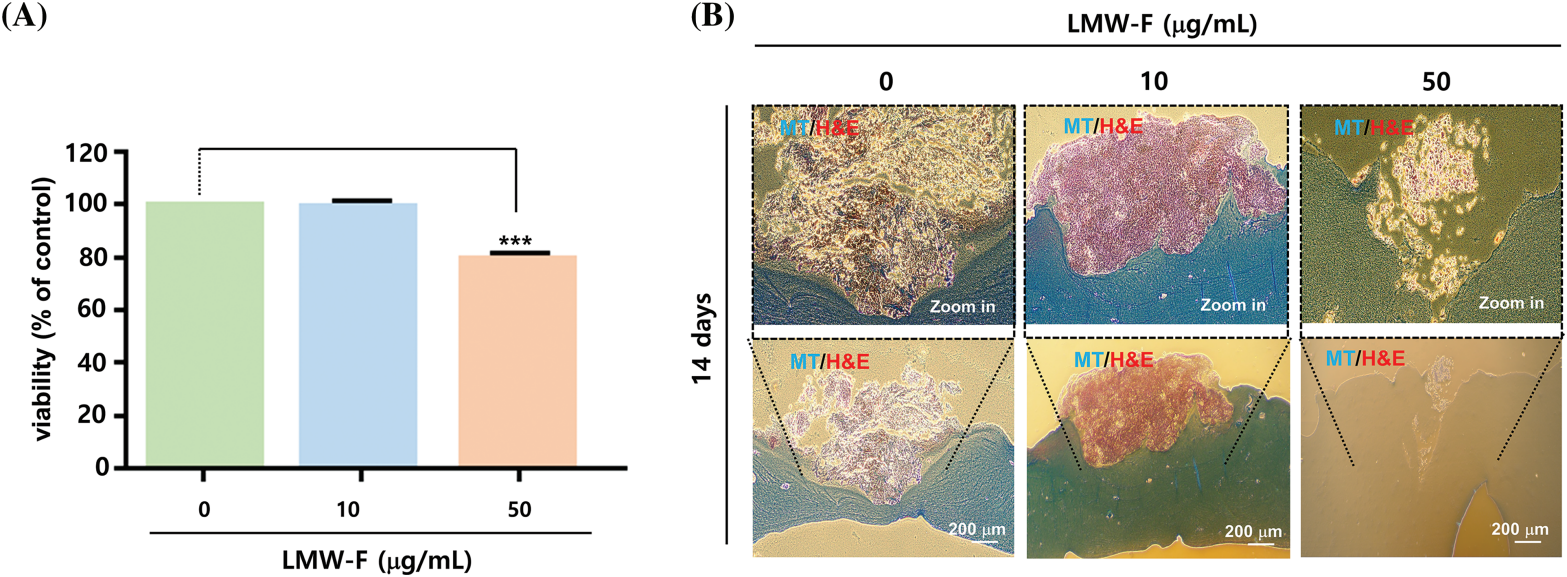
LMW-F reduces cell proliferation in 3D-PCS-PDMEs. (A) Bar graph showing the cell viability of 3D-PCS-PDMEs at various concentrations of LMW-F for 14 days, as determined by MTT assay. Data are expressed as the mean ± SD of triplicate assays, relative to control. Statistical analysis was performed using Student’s *t*-test. ***p < 0.001 *vs.* corresponding controls. (B) Histochemical analysis of LMW-F-treated 3D-PCS-PDMEs using H&E and MT staining. Scale bars indicate 200 μm.

Previous studies in our laboratory utilized 3D-PCS and PDME fragments to generate 3D-PCS-PDMEs [13,14]. To assess the effects of LMW-F (0, 10, 50 μg/mL) for 14 days (Fig. 3), we applied 3D-PCS-PDMEs. We observed distinct, dose-dependent morphological outgrowths of PDME (Fig. 3A). At a concentration of 50 μg/mL, LMW-F inhibited the morphological outgrowth of 3D-PCS-PDMEs compared to the untreated group (Fig. 3A). The WHO-RECIST measurement demonstrated a greater outgrowth of PDME in the absence of LMW-F (50 μg/mL) (Fig. 3B). We also measured the diameter and thickness of the 3D-PCS in each group. It is well-known that melanoma cells can secrete enzymes, such as matrix metalloproteinase (MMPs), that can degrade components of the extracellular matrix, including collagen [20]. As shown in Fig. 4, the diameter and thickness of the 3D-PCS-PDMEs remained unchanged in the presence of LMW-F, while the untreated group exhibited a decrease in the thickness of 3D-PCS. To further confirm these observations, we evaluated the cell viability and histological staining of the 3D-PCS-PDMEs in the presence or absence of LMW-F using MTT assays and H&E/MT staining, respectively. The results of the MTT assays and H&E/MT staining are consistent with the WHO-RECIST measurement (Fig. 5). Therefore, these observations indicate that LMW-F has potential as a candidate for inhibiting melanoma outgrowth.

### The anti-melanoma effects of LMW-F on the melanoma involve the modulation of H2B and caspase-3

**Figure 6:**
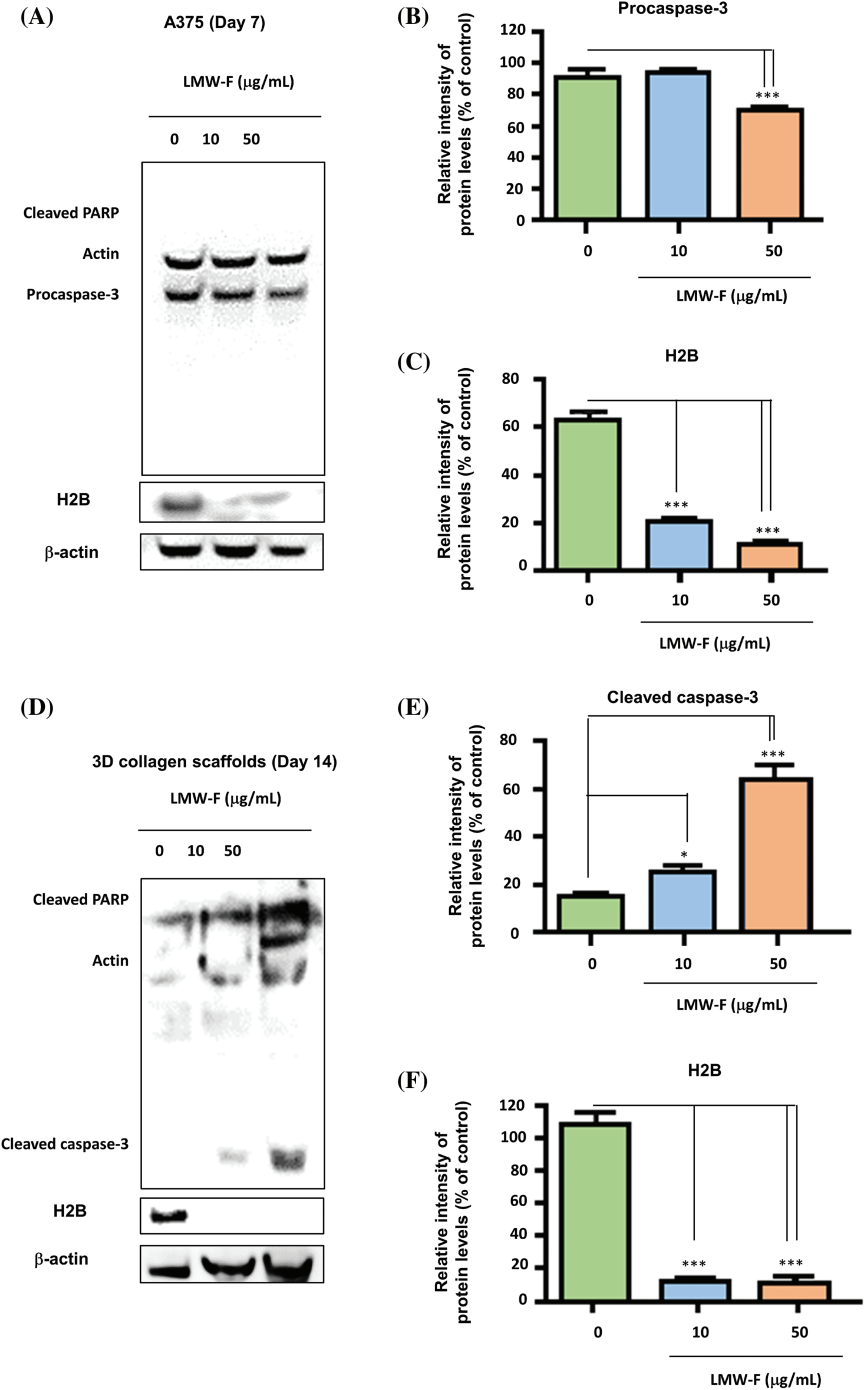
The anti-proliferative effects of LMW-F on A375 cells and 3D-PCS-PDMEs are associated with apoptotic pathways. Western blot analysis was performed to investigate the apoptotic pathway of each group, with actin serving as a loading control. (A) A375 cells treated with LMW-F in a dose-dependent manner. (B, C) The bar graphs indicate the expression levels of procaspase-3 and H2B proteins derived from quantitative analysis of the western blot images using a densitometer. (D) 3D-PCS-PDMEs treated with LMW-F in a dose-dependent manner. (E, F) The bar graphs indicate the expression levels of cleaved caspase-3 and H2B derived from quantitative analysis of the western blot images. b-actin was used as an internal control to normalize protein expression. Data are expressed as the mean ± SD of triplicate assays, relative to control. Statistical analysis was performed using Student’s *t*-test. *p < 0.05 and ***p < 0.001 versus corresponding controls.

If the anti-melanoma effects of LMW-F contribute to cell proliferation in melanoma, one of the key regulators of apoptosis, such as caspase-3, could be associated with the anti-melanoma performance of LMW-F. To investigate this hypothesis, we performed western blot analysis on A375 cells and 3D-PCS-PDMEs treated with varying concentrations of LMW-F (0, 10, 50 μg/mL) at the indicated time points (Figs. 6A–6F). In both the A375 cells and 3D-PCS-PDMEs, LMW-F (50 μg/mL) resulted in changes in the activity of caspase-3 (Figs. 6A, 6B, 6D, and 6E). Cleaved PARP was markedly observed in LMW-F-treated 3D-PCS-PDMEs compared to the untreated group (Figs. 6B and 6E). However, there was no detection of cleaved PARP and cleaved caspase-3 in LMW-F-treated A375 cells. H2B is a histone protein that plays an essential role in the process of programmed cell death, including apoptosis. Interestingly, the expression of H2B dramatically decreased in both A375 cells and 3D-PCS-PDMEs in the absence of LMW-F (Figs. 6A, 6C, 6D, 6F, and Suppl. Fig. S4). Overall, our results indicate that LMW-F may have anti-melanoma effects in A375 cells and 3D-PCS-PDMEs by changing the activity of caspase-3 and inhibiting H2B expression.

## Discussion

In this study, we explored the potential application of more soluble and bioavailable LMW-F in the treatment of advanced melanoma and investigated the precise mechanisms of LMW-F by which LMW-F exerts its anti-melanoma efficacy. In both 2D cell models and 3D-PCS-PDMEs, LMW-F showed the ability to inhibit the proliferation of melanoma cells by affecting apoptosis-related factors such as PTEN/AKT signaling pathways, caspase-3 activity, and Bcl-2 phosphorylation at Thr56, as well as by reducing Bcl-2 and H2B expression. In general, cancer cell proliferation refers to the rapid and uncontrolled growth and division of cancer cells. Compared to some other types of cancer, melanoma cells divide more quickly, with advanced melanoma exhibiting an especially great speed in spreading to other parts of the body, further complicating treatment and thus resulting in a high mortality rate [3,21,22]. Moreover, some melanomas are resistant to standard treatments such as chemotherapy and radiation, making it additionally challenging to find effective treatment options for these patients. Multi-drug resistance (MDR) in melanoma is characterized by the ability of melanoma cells to persist and proliferate despite exposure to various therapies, including chemotherapy, radiation, and immunotherapy [3,21,22]. This resistance can occur due to a variety of factors, such as genetic mutations, changes in cell signaling pathways, and alterations in the tumor microenvironment [3,21,22].

MDR in melanoma therefore remains a significant clinical challenge with limited therapeutic options [3,21,22]. Multiple mechanisms and recurrent somatic mutations have been identified that contribute to melanoma resistance, including alterations in genes such as BRAF, NRAS, and PTEN, as well as the activation of cell signaling pathways that promote cell survival and proliferation, such as the MAPK and PI3K/AKT pathways [23,24]. Notably, the PI3K/AKT pathway is an essential hub that connects various targets involved in the regulation of apoptosis, cell growth, and cellular metabolism associated with MDR [23,24]. Our findings indicated that AKT phosphorylation at Ser473 in LMW-F-treated melanoma cells gradually increased from 10 min to 1 h, followed by a decrease after 8 h, while untreated melanoma cells exhibited a marked increase in AKT phosphorylation after 8 h. Meanwhile, the phosphorylation of PTEN at Ser380 in LMW-F-treated melanoma cells displayed an expected opposing pattern to AKT phosphorylation at Ser473. These findings are consistent with several previously published studies that suggest fucoidan’s ability to suppress cancer progression through the PI3K/AKT, MAPK, and caspase pathways [25–29]. Furthermore, several studies related to fucoidan have reported how LMW-F negatively affects the PTEN/AKT signaling pathway in melanoma, similar to our observations [25–29]. Importantly, the LMW-F specifically investigated in our study, at a low concentration of 50 μg/mL, demonstrated greater anti-proliferative effectiveness against melanoma compared to fucoidan previously studied at higher concentrations of over 200 μg/mL. For example, high concentrations of fucoidan (over 200 μg/mL) induce apoptosis and prevent the proliferation of various cancer cell lines, such as breast cancer, B-cell lymphoma, T-cell lymphoma, fibroblastic sarcoma, uterine sarcoma, lung cancer, hepatocellular carcinoma, colorectal cancer, and melanoma [25–29]. These cancer cell lines also exhibit a relationship between PTEN expression and the p38 MAPK/ERK and PI3K/AKT signal pathways in the presence of fucoidan [25–29].

Fucoidan is an attractive candidate for decreasing the MDR-phenotype in cancer [27]. For instance, a prior study reported the use of fucoidan-coated coral-like Pt nanoparticles for computed tomography-guided promotion of a synergistic anti-cancer effect against the MDR breast cancer cell MCF-7 ADR both *in vitro* and *in vivo* [29]. Another study demonstrated that natural, purified fucoidan from New Zealand *Undaria pinnatifida* synergizes with the ERBB inhibitor lapatinib, enhancing the inhibition of melanoma growth [28]. More specifically, this Fucoidan, at a concentration of 1 mg/mL, doubles the cell-killing capacity of lapatinib, accompanied by a further reduction in AKT and NFκB signaling, two critical pathways involved in melanoma cell survival [28]. Importantly, the cell-killing effects of fucoidan could be enhanced by inhibiting ERBB3 with either a specific shRNA or a novel, selective ERBB3 neutralizing antibody, underscoring the pivotal role played by this receptor in melanoma [28]. Our findings that LMW-F reduces Bcl-2 phosphorylation at Thr56 and downregulates Bcl-2 expression in melanoma cells are consistent with previous studies indicating that inhibiting Bcl-2 phosphorylation can overcome resistance mechanisms [19,30–32]. For example, combining copper chelation with tetrathiomolybdate (TTM) and the Bcl-2 inhibitor ABT-263 reduces cell viability and induces apoptosis in BRAFV600E-driven melanoma cells [31]. Furthermore, TTM and ABT-263 combination therapy suppresses the growth of both naïve and MEK1/2-resistant BRAFV600E-positive melanoma xenografts [32]. In particular, Bcl-2 is associated with anti-proliferation-related signaling pathways and apoptosis pathways. For example, Bcl-2 and capase-3 are involved in the mechanisms underlying kidney injury and apoptosis caused by cadmium poisoning; 4-tert-butylphenol-induced grass carp hepatocyte injury; apoptosis; and necroptosis accompanied by changes in JNK and PARP1 [19,30].

Numerous studies have suggested that the abnormal activation of the PI3K/AKT signaling pathway contributes to the upregulation of Bcl-2 expression, leading to apoptosis-mediated MDR in various cancer therapies [22,31,33]. MDR-related cancer cells also reveal elevated levels of Bcl-2 and abnormal activation of the PI3K and AKT pathways [22,31,33]. Bcl-2 is a well-known mitochondrial anti-apoptotic factor, but the biological significance of the multi-site phosphorylation of Bcl-2 at its loop region (Ser87, Ser70, Thr69, Thr56) has remained controversial. A previous study focused on the relationship between the phosphorylation of Bcl-2 at Thr56 and leucine-rich repeat kinase 2 (LRRK2), a relationship that may be associated with Parkinson’s disease and melanoma [31,33,34]. The phosphorylation of Bcl-2 at Thr56 may act as a point of crosstalk between dysregulation of autophagy and LRRK2-mediated mitochondrial depolarization [34]. Mitochondrial dysfunction and chromatin are associated with autophagy-mediated survival in doxorubicin resistant Hela & SiHa and Hep3B & HepG2 cancer cell lines [35]. A recent study describes fucoidan as an autophagy regulator [36]. Based on these studies, we may infer the precise mechanisms underlying how LMW-F impacts the potential cooperation between the PTEN/AKT pathway, Bcl-2 expression, and caspase activity. A limitation of the present study is that it does not provide direct evidence supporting the use of LMW-F in combination with other current melanoma therapies to improve their efficacy. Further study is required to determine whether low concentrations of LMW-F can synergize with a clinically approved PI3K/AKT inhibitor or Bcl-2 inhibitor.

We generated PDMEs in a 3D-PCS to better mimic the tumor microenvironment *in vivo* [13,14]. Although 2D cell cultures have been widely used to study cancer biology and evaluate treatment, they have limitations in mimicking the complex 3D structure and microenvironment of tumors [13,14]. On the other hand, 3D cancer models provide a more physiologically relevant environment for evaluating drug efficacy [37]. Nonetheless, 3D-PCE-PDMEs are limited in tracking cellular mechanisms compared to 2D cell models. To address the limitations of both models, we explored the effects of LMW-F in both 2D and 3D melanoma models. The *in vitro* 2D cell model in our study showed the precise mechanism in LMW-F through the AKT-PTEN-Bcl-2 network, while the 3D-PCS-PDME model revealed in more detail the toxicity, side effects, and physiological relevance of LMW-F. Understanding the mechanisms underlying melanoma resistance, such as alterations in genes and signaling pathways, including the MAPK and PI3K/AKT pathways, is crucial to tackling the MDR of melanoma [22,24,26,29–36]. These findings suggest that LMW-F may be useful in treating melanoma by inhibiting proliferation through modulation of PTEN-AKT-Bcl-2 networks.

In conclusion, our study demonstrated that LMW-F inhibits the proliferation of melanoma cells by targeting the PTEN/AKT signaling pathway and Bcl-2 phosphorylation. It also showed potential anti-melanoma effects by negatively impacting the outgrowth of 3D-PCS-PDMEs as well as modulating caspase-3 activity and H2B expression. Further research is needed to investigate the ability of LMW-F to overcome melanoma resistance mechanisms in greater detail, particularly in combination with other therapies.

### Statistical analysis

Student’s *t*-tests (for comparisons of two groups) or a one-way analysis of variance (ANOVA, for comparisons of three or more groups) followed by Tukey *post hoc* tests were used for the statistical analyses. SPSS software ver. 17.0 (SPSS, Chicago, IL, USA) was used. A value of *p* < 0.05 was considered significant. Data are compressed as means ± standard error of the mean (SEM). Data analysis was carried out using GraphPad Prism software (GraphPad Software Inc. Sandie go, CA, USA). *p < 0.05–0.01, **p < 0.01–0.001, and ***p < 0.001 *vs*. corresponding controls. All errsssor bars represent the standard deviation of three or more biological replicates.

## Supplementary Materials

Figure S1 The effects of LMW-F on CCD-986 cells. (A) Confocal live images show dose-dependent effects of LMW-F treatment on cell proliferation in CCD-986 cells, independently indicating live cells (green) and dead cells (red). Scale bars represent 100 µm. (B) Dose-dependent cell viability of LMW-F-treated CCD-986 cells. (CB) Dose-dependent cell proliferation of LMW-F-treated CCD-986 cells. CCD-986 cells were treated with LMW-F (0 to 50 µg/ml) and incubated for 24 h to assess cell viability or 72 h to evaluate cell proliferation. The bar graph represents triplicate assays and is expressed as a percentage of the untreated group. Data is presented as the mean ± standard deviation (SD) and was analyzed using Student’s t-test. *** p < 0.001 versus corresponding controls.

Figure S2 The quantification of phosphorylation levels of ERK, JNK, p-38, and Bcl-2 at Ser70 in A375 cells with or without LMW-F at the indicated time points. The line graphs show the phosphorylation levels of ERK, JNK, p38, and Bcl-2 at Ser70 based on quantitative analysis of the western blot images (control, green circle; LMW-F, blue box). Data are expressed as the mean ± SD of triplicate assays, relative to control. Statistical analysis was performed using Student’s t-test. *** *p* < 0.001 versus corresponding controls.

Figure S3 Western blot raw data of Fig. 2

Figure S4 Western blot raw data of Fig. 6

## Data Availability

All data supporting the findings of this study are available from the corresponding author (phdjeongym12@tukorea.ac.kr) upon reasonable request.

## Abbreviations

3D-PCS: 3D-printed collagen scaffold
LMW-F: Low-molecular-weight fucoidan
MT: Masson’s trichrome
MDR: Multi-drug resistance
PDME: Patient-derived melanoma explant
3D-PCS-PDME: Patient-derived melanoma explants in a 3D-printed collagen scaffold

## Supplementary Materials

The supplementary materials is available online at https://doi.org/10.32604/or.2023.044362.
