# Supplementary material for: Low-molecular-weight fucoidan inhibits the proliferation of melanoma via Bcl-2 phosphorylation and PTEN/AKT pathway
Source: Oncol Res. 2023 Dec 28;32(2):273–82. doi: 10.32604/or.2023.044362 (PMC10765131; doi:10.32604/or.2023.044362)
Supplement: Supplementary file 5 [file OncolRes-32-44362-s001.docx]

**Figure S1. The effects of LMW-F on CCD-986 cells.** (A) Confocal live images show dose-dependent effects of LMW-F treatment on cell proliferation in CCD-986 cells, independently indicating live cells (green) and dead cells (red). Scale bars represent 100 µm. (B) Dose-dependent cell viability of LMW-F-treated CCD-986 cells. (CB) Dose-dependent cell proliferation of LMW-F-treated CCD-986 cells. CCD-986 cells were treated with LMW-F (0 to 50 µg/ml) and incubated for 24 h to assess cell viability or 72 h to evaluate cell proliferation. The bar graph represents triplicate assays and is expressed as a percentage of the untreated group. Data is presented as the mean ± standard deviation (SD) and was analyzed using Student's t-test. ****p < 0.001* versus corresponding controls.

**Figure S2. The quantification of phosphorylation levels of ERK, JNK, p-38, and Bcl-2 at Ser70 in A375 cells with or without LMW-F at the indicated time points.** The line graphs show the phosphorylation levels of ERK, JNK, p38, and Bcl-2 at Ser70 based on quantitative analysis of the western blot images (control, green circle; LMW-F, blue box). Data are expressed as the mean ± SD of triplicate assays, relative to control. Statistical analysis was performed using Student’s *t*-test. *** *p* < 0.001 versus corresponding controls.

Supplementary Table 1. Validated primary antibodies for western blot analysis.

| Antibody | Dilutions | Solvent | Supplier | Molecular Weight |
| --- | --- | --- | --- | --- |
| pAKT (Ser473) | 1:1000 | 5% BSA in TBST | Cell Signaling | 60 kDa |
| p-Bcl-2 (Ser70) | 1:1000 | 5% BSA in TBST | Santa Cruz | 28 kDa |
| p-Bcl-2(Thr56) | 1:1000 | 5% BSA in TBST | Cell Signaling | 28 kDa |
| Bcl-2 | 1:1000 | 5% BSA in TBST | Santa Cruz | 28 kDa |
| p-PTEN(Ser380) | 1:1000 | 5% BSA in TBST | Cell Signaling | 54 kDa |
| p-ERK | 1:1000 | 5% BSA in TBST | Cell Signaling | 42, 44 kDa |
| p-p38 | 1:1000 | 5% BSA in TBST | Cell Signaling | 43 kDa |
| p-JNK | 1:1000 | 5% BSA in TBST | Cell Signaling | 46, 54 kDa |
| H2B | 1:1000 | 5% BSA in TBST | Santa Cruz | 19 kDa |
| Caspase-3 | 1:1000 | 5% BSA in TBST | Santa Cruz | 37 kDa |
| actin | 1:5000 | 5% BSA in TBST | Santa Cruz | 42 kDa |
| Apoptosis western blot cocktail | 1:1000 | 5% BSA in TBST | Abcam | Cleaved-PARP, 89 kDa; procaspase-3, 32 kDa; actin, 42 kDa; |

**Figure S3. Western blot raw data of Fig. 2**

**Figure S4. Western blot raw data of Fig. 6**
